# Levels of Resistance to Pyrethroid among Distinct* kdr* Alleles in* Aedes aegypti* Laboratory Lines and Frequency of* kdr* Alleles in 27 Natural Populations from Rio de Janeiro, Brazil

**DOI:** 10.1155/2018/2410819

**Published:** 2018-07-11

**Authors:** Luiz Paulo Brito, Luana Carrara, Rafael Maciel de Freitas, José Bento Pereira Lima, Ademir J. Martins

**Affiliations:** ^1^Laboratório de Fisiologia e Controle de Artrópodes Vetores, Instituto Oswaldo Cruz, FIOCRUZ, Rio de Janeiro, Brazil; ^2^Laboratório de Entomologia, Instituto de Biologia do Exército, Rio de Janeiro, Brazil; ^3^Laboratório de Transmissores de Hematozoários, Instituto Oswaldo Cruz, FIOCRUZ, Rio de Janeiro, Brazil

## Abstract

**Background:**

Several mutations in voltage gated sodium channel (Na_V_) have been identified in* Aedes aegypti* populations worldwide. However, only few are related to knockdown resistance to pyrethroids, most of which with variations in the 1016 and 1534 Na_V_ sites. In Brazil, at least two* Na*_*V*_ alleles are known: Na_V_R1, with a substitution in the 1534 (1016 Val^+^ + 1534 Ile^*kdr*^) and Na_V_R2, with substitutions in both 1016 and sites (1016Ile^kdr^ + 1534Cys^*kdr*^). There is also the duplication in the* Na*_*V*_ gene, with one copy carrying the substitution Ile1011Met, although its effects on pyrethroid resistance remain to be clarified. Our goals in this study were (1) to determine the role of each* kdr *Na_V_ allele and the duplication on pyrethroid resistance and (2) to screen the frequency of the* kdr* alleles in 27 several natural* Ae. aegypti* populations from the metropolitan region of Rio de Janeiro.

**Methods:**

Pyrethroid resistance was evaluated by a knockdown time (*Kd*T) assay, an adaptation of the WHO test tubes with paper impregnated with deltamethrin. We used laboratory-selected* Ae. aegypti* lineages: R1R1 and R2R2 (homozygous for the* kdr *Na_V_R1 and Na_V_R2 alleles, respectively), Dup (with duplication in the* Na*_*V*_ gene), Rockefeller (the susceptibility reference control), and F1 hybrids among them. Genotyping of both 1016 and 1534 Na_V_ sites was performed in 811* Ae. aegypti* sampled from 27 localities from Rio de Janeiro (17), Niterói (6) and Nova Iguaçu (4) cities, Rio de Janeiro State, Brazil, with a TaqMan real time PCR approach.

**Results:**

The laboratory lineages R1R1, R2R2, and R1R2 were the only ones that needed more than 60 minutes to* knock down* all the insects exposed to the pyrethroid, being the* Kd*T R2R2 > R1R2 > R1R1, corroborating the recessive nature of the* kdr* mutations. Frequency of* kdr* alleles Na_V_R1 and Na_V_R2 in field-caught mosquitoes varied from 0 to 52% and 43 to 86%, respectively, evidencing high levels of “resistant genotypes” (R1R1, R1R2, and R2R2), which together summed 60 to 100% in* Ae. aegypti* populations from Rio de Janeiro.

**Conclusions:**

The Na_V_R1 and Na_V_R2* kdr* alleles confer resistance to the pyrethroid deltamethrin in homozygotes and R1R2 heterozygotes, being the R2R2 most resistant genotype. The allele containing duplication in the Na_V_ gene, with a mutation in the 1011 site, did not confer resistance under the tested conditions. The frequencies of the “resistant genotypes” are elevated in* Ae. aegypti* natural populations from Rio de Janeiro.

## 1. Introduction

*Aedes aegypti* is the primary vector of dengue, chikungunya, and Zika virus in tropical and subtropical regions of the globe. The incidence of dengue cases has dramatically increased in the last decade, with an estimation of 390 million dengue infections per year [1], summed with the recent re-emergence of chikungunya and Zika. New-born malformations and neurological complications associated with Zika led the World Health Organization (WHO) to declare the “Public Health Emergency of International Concern” in 2016 [2]. This situation worsened with a strong concern regarding a potential reurbanization of yellow fever virus in Brazil, which have killed more than 200 people in rural municipalities from July 2017 to March 2018, i.e., 8 months [3]. This scenario reinforces the need to strengthen vector control measures to mitigate disease transmission.

New vector control strategies to reduce* Ae. aegypti* population density below a critical threshold have been proposed, which are expanding to vast open field application tests, with support of local communities, governments, and stimulation by WHO [4]. The dissemination of transmission blocking mosquitoes carrying* Wolbachia* and the release of transgenic-based sterile mosquitoes (RIDL) are among the most promising approaches developed so far [5, 6]. On the other hand, the employment of insecticides will persist for a long time as a prime strategy for rapidly reduction on mosquito density, especially during an outbreak. In this sense, it is important that these compounds are efficient over target populations. Among the classes of insecticides recommended by the World Health Organization Pesticide Scheme (WHOPES), pyrethroids are the most employed against* Aedes* mosquitoes since they provoke the fast-acting* knockdown* effect, are cheaper, and cause less nuisance to householders indoor. However, the excessive and uncontrolled employment of insecticides has been selecting* Ae. aegypti* resistant populations worldwide [7, 8]. The principal physiological mechanisms selected for pyrethroid resistance are related to increase in the expression profile of metabolic enzymes, especially* cyp* P450 genes of the multifunction oxidases class, and point mutation in its target site, the voltage gated sodium channel (Na_V_) [8, 9].

There are several mutations in insect* Na*_*V*_ genes conferring resistance to pyrethroid, with the L1014F* kdr* substitution being the most common, conserved among distinct insect orders. This happens since there are few modifications permitted in the highly conserved* Na*_*V*_ gene, which responds for a central role in the neuron physiology of animals [10]. Additionally, the same* kdr* mutations may have multiple origins in a species, as evidenced for insects such as* M. domestica* [11] and* An. gambiae* [12]. In* Ae. aegypti*, however, the L1014F* kdr* mutation is not found due to codon constraint, to which the simultaneous selection of two mutations in the same codon would be necessary, which is unlikely to happen [13]. Besides the fact that some* kdr* mutations are specific for some species, in* Ae. aegypti* this number is uniquely high [14]. Other mutations are found in several positions of the* Ae. aegypti *Na_V_, where the relationship with resistance to pyrethroid is better described to 1016 (Val to Ile in Americas and Africa or Gly in Asia and Middle Eastern) and 1534 (Phe to Cys) Na_V_ sites [15–18]. In* Ae. aegypti* Latin American populations, the allele containing a mutation in the 1534 site is widely distributed; meanwhile the alleles with mutations in both 1016 + 1534 sites are increasing in frequency and dispersing [17, 19]. Considering these two sites, in Brazil there is evidence of three alleles, here called Na_V_S (1016 Val^+^ + 1534Phe^+^), Na_V_R1 (1016Val^+^ + 1534Cys^*kdr*^), and Na_V_R2 (1016Ile^*kdr*^ + 1534Cys^*kdr*^) [19, 20]. The I1011M is another substitution found in* Ae. aegypti* populations from Latin American and is involved in a gene duplication event [21]. Although it was proved to alter the sodium channel sensitivity to pyrethroids [22], its actual role in resistance is controversial in natural populations where other* kdr* mutations occur [23, 24]. The S989P substitution (together with V1016G and F1534C) also plays an important part for pyrethroid resistance, but its distribution seems to be restricted to Middle East/Asia [15]. Genotyping of known* kdr* single nucleotide polymorphisms (SNPs) in susceptible and resistant individuals from laboratory-selected lineages as well as natural populations and electrophysiological studies evidencing altered Na_V_ sensibility to pyrethroids have been corroborating the hypothesis of such SNPs with knockdown resistance [14]. Electrophysiological assays are important to determine the role of only and combined mutations in the sensibility to pyrethroids. Not less important is to evaluate the whole organism, making use of laboratory lines with homogeneous genetic backgrounds.

Here we evaluated the role of* kdr* mutations occurring in Brazilian* Ae. aegypti* populations in response to the pyrethroid deltamethrin, based on bioassays with laboratory-selected lines without interference of other known mechanisms. In addition, the frequency of kdr mutations was established for* Ae. aegypti* natural populations from 27 localities in Rio de Janeiro State, the most touristic city from South America and likely the port of entry of dengue virus, serotypes 1, 2, and 3 in Brazil [25, 26].

## 2. Methods

### 2.1. Laboratory Lineages

Rockefeller is an* Ae. aegypti* lineage reference for physiology experiments and constantly employed as a baseline of insecticide susceptibility [27]. In our laboratory, it has been continuously maintained since 1999 [28]. Rockefeller is homozygous for the Na_V_S allele (1016 Val^+^ + 1534 Phe^+^). The lineage here called R2R2 is the same Rock-kdr previously described in [29], homozygous for the allele Na_V_R2 (1016 Ile^*kdr*^ + 1534 Cys^*kdr*^) and maintained in the laboratory since 2012. The lineage Dup does not harbour the* kdr* mutations in the 1016 and 1534 Na_V_ sites but a duplication in the* Na*_*V*_ gene and a substitution at the 1011 site, keeping both variations 1011 Ile and 1011 Met [21].

For obtaining the R1R1 lineage, we used insects maintained in the laboratory, originally collected at Santarem, PA, Brazil, a city with high frequency of the Na_V_R1 allele and no register of Na_V_R2 [19, 30]. We set up groups of one male with three virgin females, maintained together for three days in 50 mL conical plastic tubes, under the insectary conditions. Afterwards, males were removed and genotyped for the Na_V_ 1534 site (see below); meanwhile females were offered to blood meal on anesthetized mice and three days after were individually induced to egg-laying in 6 cm Petri dishes covered with a wet filter paper, as described elsewhere [29]. After egg-laying, females were also genotyped. Eggs from both parents revealed as R1R1 were induced to hatch, resulting in a total of 86 larvae that gave origin to this first R1R1 lineage.

In order to homogenize the genetic background of the* kdr* lineages, we further backcrossed that R1R1 new lineage with the previous established R2R2 [29]. To accomplish that, we first mixed R1R1 males with R2R2 females, obtaining an R1R2 offspring (F1). Males from this F1 (R1R2) were then backcrossed with R2R2 females, resulting in a F2 with the genotypes R1R2 and R2R2, in an expected 1:1 proportion. Groups of one F2 male with two R2R2 females were set up in conic tubes and carried out similarly as above. Males were genotyped and females were induced to lay eggs (F3). The F3 eggs resulting from R1R2 males were used for producing the next generation. This procedure was repeated for two more generations until F5. Then, similar groups were formed, now with both males and females from this F5. The F6 resulting eggs used for moving forward belonged to the offspring of male and female both genotyped as R1R2, among which 25% was expected to be R1R1. Finally, new groups were set up among the F6 adults. From the F7 resulting eggs, those originated from R1R1 parental were used to finally establish the new R1R1 lineage (supplementary figure S1).

Therefore, a part of the* Na*_*V*_ locus, the original homozygote colonies (Rockefeller, R1R1 and R2R2) had more similar genetic background, with exception of the Dup lineage with duplication in the* Na*_*V*_, which was not backcrossed with any of these lineages.

### 2.2. Bioassays

An adaptation of WHO test tubes bioassays [31] was performed with* Ae. aegypti* females exposed to papers impregnated in the laboratory with the pyrethroid deltamethrin at 1.7 g/cm^2^ (0.034 % solution). Deltamethrin (Sigma-Aldrich) was dissolved to a stock concentration at 10% in acetone and then diluted to an intermediate solution at 1% in silicone (Dow Corning), following a new dilution to a work solution at 0.034 % (340 mg/L) also in the silicone. A total of 840 *μ*L of this work solution was then pipetted over a 12x14 cm^2^ filter paper sheet (Whatman Grade 1), with the help of an electronic multichannel pipette (Eppendorf) and a frame, which oriented the dispersion of 5 *μ*L at each 96 equidistant spots. At all batches of impregnation, some papers were filled only with silicone as negative control. The paper sheets were air-dried for two days before their use in the bioassays. The bioassays proceeded as indicated [31]. Around 20 3-5 days old* Ae. aegypti* females were transferred to the resting tube and then gently blown into the respective test tube, where the knockdown was followed for up to 2 h, in intervals of 2 or 5 minutes. Each lineage was assayed with four tubes, in at least two independent times (females resulted from distinct batches of eggs and papers from different impregnation lots). Probit analysis [32] was performed in order to infer the time necessary to knockdown 95% of the individuals of each lineage (KdT_95_). The resistance ratio (RR_95_) was taken by the quotient between the KdT_95_ of a given lineage with the Rockefeller's.

### 2.3. *Aedes aegypti* from Rio de Janeiro State (RJ)

We took advantage of a wide sampling previously performed in 2012 in 27 localities in RJ metropolitan area, comprising three municipalities: Rio de Janeiro city (Tubiacanga, Valqueire, Urca, Olaria, Gamboa, Cajú, Pavuna, Méier, Grajaú, Paquetá, Vaz Lobo, Jardim Guanabara, São Cristóvão, Humaitá, Rio Comprido, Rio das Pedras, and Taquara neighbourhoods), Niterói City (Jurujuba, Itacoatiara, São Franciso, Fonseca, Ponta D'Areia, and Piratininga neighbourhoods), Nova Iguaçu City (Cabuçu, Cerâmica, and Moquetá neighbourhoods), and Belford Roxo City (Heliópolis neighbourhood) [33]. Briefly, mosquito eggs were collected during three consecutive weeks using 60 ovitraps in a grid of 500 x 500 m^2^ per neighbourhood. Thus, we believe our sample was representative of the genetic variation presented in each neighbourhood. Ovitraps' paddles were brought to the lab, eggs were hatched, and larvae were reared until the adult stages, when the DNA was extracted.

### 2.4. kdr Genotyping

DNA was extracted from individual insects tittered in 200 *μ*L squishing buffer (10 mM Tris-HCl pH 8.2, 2 mM EDTA, and 0.2 % Triton X-100) and 0.2 mg/L Proteinase K (Promega), as described elsewhere [34]. A customized TaqMan genotyping assay (Thermo Fisher Scientific) was employed for 1016 (Val^+^ and Ile^*kdr*^) and 1534 (Phe^+^ and Cys^*kdr*^), independently for each Na_V_ site [20]. Reactions were performed in 10 *μ*L containing 1*μ*L DNA, 1X TaqMan Genotyping Master Mix (Thermo Fisher Scientific), the mix of primers and probes Custom TaqMan SNP Genotyping Assay (1X for Val1016Ile, AHS1DL6 and 0.5X for Phe1534Cys, AHUADFA, Thermo Fisher Scientific), and H_2_O q.s. 10 *μ*L, with the thermocycling program in accordance with the manufacturer's instructions in QuantStudio 6 qPCR equipment (Thermo Fisher Scientific).

For genotyping variations in the 1011 site (Ile or Met) we employed an allelic-PCR approach, in which the specific products were detected through a dissociation curve analysis after the amplification reaction, as described elsewhere [13, 21]. Reactions contained 1X Sybr Green Master mix (ThermoFischer), 20 ng DNA, 0.24 *μ*M primer 1011_forward 5′-GTCCTGTATTCCGTTCTTTTT-3′common to both sequences, and 0.12 *μ*M of each two specific primers: 1011_Ile_reverse 5′-[long tail]-TACTTACTACTAGATTTGCC-3′ and 1011_Met_reverse 5′-[short tail]-TACTTACTACTAGATTTACT-3′ and H_2_O q.s. 12.5 *μ*L. The specificity laid on the 3′-end of the specific primers and the discrimination of the amplicons was possible due to a GC tail at the 5′-end of both specific primers, however with distinct sizes: short [GCGGGC] and long [GCGGGCAGGGCGGCGGGGGCGGGGCC], providing TM of 77°C and 82°C, respectively, in a dissociation curve analysis. For more details about this method, please check Saavedra-Rodriguez et al 2007 [13]. The thermocycling program consisted of 35 cycles (denaturation 94°C/ 30′′, annealing 57°C/ 1′and polymerase extension 72°C/ 45′′), followed by the standard melt curve analysis in a QuantStudio 6 qPCR equipment (Thermo Fisher Scientific).

The 95% Confidence Intervals (CI95%) of the allelic frequencies were calculated using the exact binomial approximation (http://www.biostathandbook.com/confidence.html). Comparisons among genotypic frequencies pairs were performed with exact G test and Fisher's method, with default Markov chain parameters by Genepop version 4.2, online version (http://genepop.curtin.edu.au).

## 3. Results

### 3.1. *Ae. aegypti* Lineages and Bioassays

**Figure 1:**
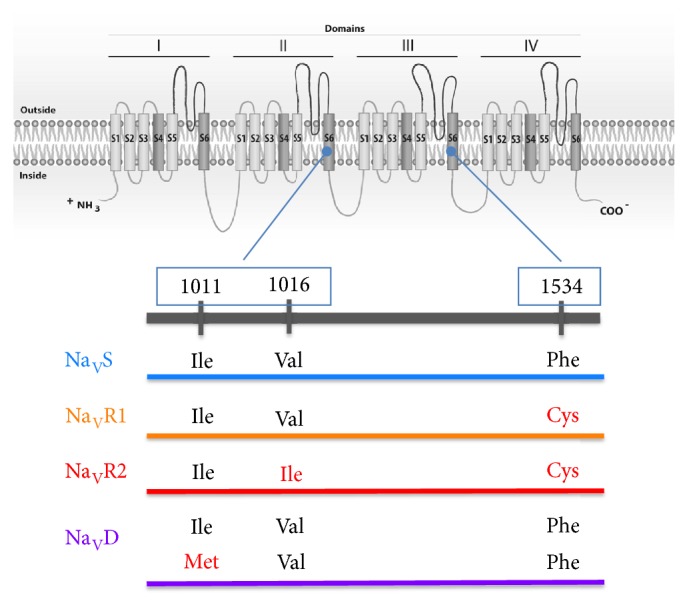
**Voltage gated sodium channel (Na**_**V**_ **)* kdr* alleles of* Aedes agypti* populations from Brazil.** Schematic representation of the Na_V_, with its four domains, each with six hydrophobic segments. The* kdr* sites 1011 and 1016 are in the IIS6 segment, while the 1534 site lies in the IIIS6. Each haplotype is represented by means of the variation at each* kdr* site, where wild-type and* kdr* aminoacids are indicated in black and red, respectively. The colours of the alleles are the same in the following figures.

The results of 1016 and 1534 SNPs were merged to constitute the genotypes as presented in Table 1 and were composed by the three alleles: Na_V_S (1016 Val^+^ + 1534Phe^+^), Na_V_R1 (1016Val^+^ + 1534Cys^*kdr*^), and Na_V_R2 (1016Ile^*kdr*^ + 1534Cys^*kdr*^). Therefore, all insects 1016 (Val/Ile) + 1534 (Phe/Cys) were considered SR2 (see Table 1). There was the additional Na_V_D, which represents a duplication in the* Na*_*V*_ gene, similar to Na_V_S in relation to 1016 and 1534 sites, however with a copy 1011Ile^+^ and another 1011 Met^*kdr*^ (Figure 1).

**Figure 2:**
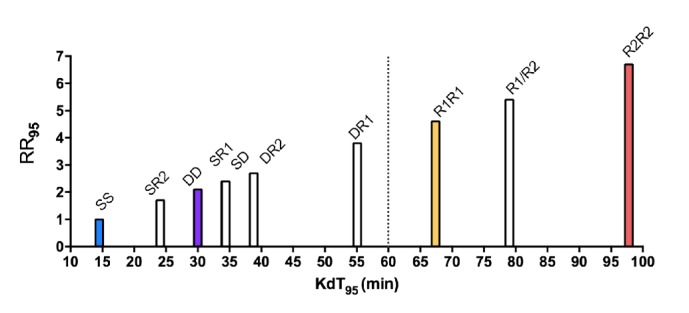
**The knockdown time (*Kd*T) profile of* Aedes aegypti* laboratory lineages with distinct Na**_**V**_ **genotypes**. The abscissa and ordinate in the graph indicate respectively the time in minutes for knockdown 95% of the insects (*Kd*T_95_) from the respective lineages and their resistant ratio (RR_95_), considering Rockefeller (SS) as reference.

The RR_95_ based on Rockefeller (SS) of each lineage and their hybrids are graphically represented in Figure 2 and the KdT_95_ values expressed in minutes are detailed in the supplementary Table S1. The only insects still flying after 60 minutes of exposition to deltamethrin belonged to the genotypes R1R1, R1R2, and R2R2, i.e., homozygous for the* kdr *Na_V_R1 and Na_V_R2 alleles and the heterozygous hybrid. Excluding R1R2, the RR_95_ of hybrids genotypes (i.e., with Na_V_S or Na_V_D alleles) varied between 1.7 and 3.8, confirming the recessive trait of* kdr* mutations regarding resistance to the pyrethroid deltamethrin.

The R2R2 lineage, homozygous for the* kdr *Na_V_R2 allele, was the most resistant (RR_95_ = 6.7). R2R2 individuals were 1.5-fold more resistance than R1R1, considering their KdT_95_. An exposition of 30 minutes to the insecticide was sufficient to knockdown 95% of Dup females.

### 3.2. *Kdr* Genotyping of* Ae. aegypti* Natural Populations from Rio de Janeiro State (RJ)

**Figure 3:**
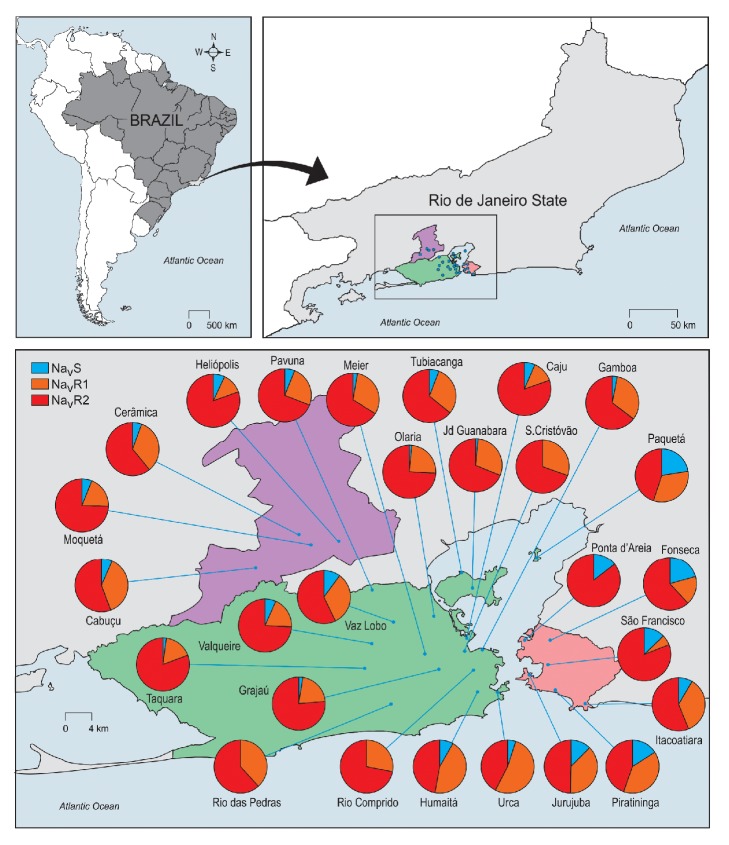
**Frequencies of* kdr* alleles, considering 1016 and 1534** **N** **a**_**V**_ **sites, of* Aedes aegypti* from Rio de Janeiro State.** Localities are indicated in the map. The shadowed area in green, pink, and grey represent the regions Rio, Niterói, and Baixada, respectively.

The three Na_V_ alleles Na_V_S, Na_V_R1, and Na_V_R2 were observed among the total of 811 genotyped insects (Figure 3). Number of samples evaluated ranged from 12 (Jurujuba, Niterói) to 73 (Cerâmica, Nova Iguaçu). In total average, the Na_V_R2* kdr* allele was the most frequent (65.4%), followed by Na_V_R1 (27.5%) and Na_V_S (7.2%). Out of the 27 localities, only Urca presented the Na_V_R1 allele with the highest frequency (52,5%) (Supp Table S1). The wild-type Na_V_S was far the less frequent, ranging from 0 (Humaitá, Rio das Pedras and São Cristóvão, Rio de Janeiro) to 22.5% (Paquetá island, Rio de Janeiro), except Fonseca (Niterói) where the Na_V_S frequency (20.6%) was higher than the Na_V_R1 allele (17.6%). Interestingly, when pooling neighbourhood's data to their respective regions, Na_V_S frequency was higher in Niterói (14,2%) than in Rio (4,0%), a significant difference, by considering that there is no overlapping among theirs IC95% (supp material S2). Paquetá was removed from this analysis, since it is an island distant from both Rio and Niteroi offshores. These cities are separated by the Guanabara Bay, connected by a 13 Km bridge and ferry boats.

The R2R2 genotype, which would potentially account for higher levels of resistance to pyrethroids, was the most frequent genotype (median 50.0%), and the “resistant genotypes” (R1R1, R2R2, and R1R2) together reached a median of 88.4% among* Ae. aegypti* from the sites evaluated (Table 2). The localities with the lowest frequency of the “resistant genotypes” were Paquetá island (60%), followed by five out of the six neighbourhoods evaluated from Niterói (64.7% in Fonseca-75.7% in Piratininga).

The genotypic frequencies among regions (Baixada, Rio, Niteroi, and Paquetá island) did not differ significantly between Rio and Baixada, as well as between Niteroi and Paquetá (both exact G test P>0.05). In their turn, Rio/Baixada highly differed from Niteroi/Paquetá (see Table 3).

## 4. Discussion

Here we evaluated the difference in insecticide resistance levels to the pyrethroid deltamethrin in* Ae. aegypti* laboratory lines with distinct* kdr* mutations introgressed from the field and free of other known resistance mechanism. Several records of increased levels of resistance to pyrethroid, in parallel with dissemination of* kdr* alleles in natural vector populations, have been released in the last decade, as an indirect correlation between* kdr* mutation and pyrethroid resistance in* Ae. aegypti* [8, 35]. Laboratory selection pressure experiments with insecticide have also corroborated this correlation, when* kdr* frequencies have increased toward fixation [13]. In addition, electrophysiological tests based on natural populations as well as samples that undergone site directed mutagenesis confirmed that* kdr* mutations found in* Ae. aegypti* alter sensibility to pyrethroids [36, 37]. However, to our knowledge, this is the first study* in vivo* that evinces the importance of such mutations in homogeneous* kdr* laboratory lines of* Ae. aegypti*, except for the* kdr* locus, i.e., with minimum interference of other possibly selected mechanisms, as well as of pleiotropic effects that might respond differently to each distinct genetic background.

WHO Pesticide Evaluation Scheme (WHOPES) recommends discriminating concentrations of insecticides for the impregnated paper tests [38]. The most recent WHOPES plan for detecting and monitoring IR in* Aedes* aegypti recommended 0.03% as a discriminating concentration of deltamethrin [39], to where mortality is recorded 24 h after 1 h of exposition to the insecticide. Mortality under 90% indicates resistance of the evaluated population, as long as at least 100 mosquitoes were tested. This is a qualitative dose-diagnostic test, good for determining the susceptibility status of a given population although not suitable for comparing levels of resistance among populations. For this matter, a quantitative dose-response test is indicated, in which the mortality rates over a range of insecticide concentrations generate the lethal concentrations (LC) of each population. In turn, LC produces the resistance rations (RR), based on a reference lineage [40]. However, this sort of test requires a large number of insects compared to the dose-response approach. Here we applied a WHO-like dose-diagnostic test with papers impregnated on our own. Nevertheless, instead of evaluating mortality 24 h after 1h of exposition, we followed the knockdown rate for up to 2 h. With this record over the time, a semiquantitative analysis was performed by extracting the RR of the populations, in this case based on their time of knockdown. It is worth noting that this knockdown time RR displays a different scale, generally shorter than those produced by truly qualitative dose-response tests.

We employed a very efficient TaqMan assay for the rapid genotyping of the* kdr* alleles present in* Ae. aegypti* Latin American populations. Our previous allele specific PCR, although useful, generally needed constant adjustments in the number of cycles and/or the concentration of specific primers according to different thermal-cycle machinery or PCR kit employed [19, 21]. Sometimes, unspecific shallow amplification also occurred, pointing to the need of additional confirmatory reactions. The present TaqMan assay renders the genotyping process more clear and straightforward. However, one must be aware that any genotyping assay will only reveal the specific alleles available in that assay. For instance, instead of a Val/Ile mutation found in* Ae. aegypti* from Latin American, the* kdr* mutation in the 1016 Na_V_ site of Asian populations is a Val/Gly [14], to which the assay employed here is unable to detect. This is also true for other potential mutations initially under low frequencies. Therefore, these allele specific methods should only be used to evaluate genotypic frequencies of populations with a previously well-explored genetic background of the target genes.

The physiological importance of the* kdr* mutations and altered sensitivity to pyrethroids have been evaluated with mutant insect* Na*_*V*_ gene presenting punctual or combined mutations in* Xenopus* oocites heterologous expression system, followed by electrophysiological assays [22]. Such assays, employing direct mutagenesis of* AaNa*_*V*_ cDNA, demonstrated that the V1016I mutation alone (which we would call Na_V_R3 allele) did not alter the channel sensitivity to pyrethroids, while F1534C* kdr* mutation (Na_V_R1 allele) reduced the channel affinity of pyrethroid type I (permethrin), but not type II (deltamethrin) [37]. In agreement, this same mutation reduced the affinity to type I but not to type II pyrethroids in the cockroach* Blatella germanica* channel [41]. However, herein we found that although Na_V_R1 conferred lower level of resistance than Na_V_R2, R1R1 insects were resistant to deltamethrin, a type II pyrethroid. The Na_V_R2 allele used to be absent in some deltamethrin resistant populations from the Northeast of Brazil, where the Na_V_R1 was found in high frequencies [19]. More recent samplings evidenced that the Na_V_R2 is disseminating and increasing in frequency also through those areas [20, 30, 42]. Vera-Malof et al. [17] proposed that the Na_V_R1 had emerged first, conferring low levels of resistance to pyrethroids, and then the V1016I arose from that allele, originating the Na_V_R2. Na_V_R2 would have been rapidly selected and dispersed, by conferring higher levels of resistance to pyrethroids. The possible Na_V_R3 (1016Ile^*kdr*^ + 1534Phe^+^) has not ever been evidenced in Brazilian* Ae. aegypti* populations and therefore was not considered in our analysis. Indeed, we did not find any SR3, R2R3, and R3R3 individual.

In the house fly* Musca domestica,* the relationship of three Na_V_ alleles with resistance to pyrethroid was investigated, by evaluating the susceptibility of congenic strains and their hybrids to a range of several pyrethroid compounds. The double mutant* super-kdr* allele (M918T + L1014F) conferred more resistance than the classical* kdr* (L1014F), which in its turn conferred more resistance than the* kdr-his* (L1014H). The heterozygotes kdr/super-kdr and super-kdr/kdr-his presented intermediate resistance between the homozygous, characterizing an incomplete recessive inherence partner for these* M. domestica *Na_V_ alleles [43].* Ae. aegypti* field populations from Malaysia showed increased resistance when presenting* kdr* mutation in both 1016 and 1534 Na_V_ sites [44]. In that case, however, substitution in the 1016 site was Val to Gly, as common in Middle Eastern and Asian populations [15, 45, 46]. Here a similar partner was observed in* Ae. aegypti*. The double mutant* kdr* allele Na_V_R2 conferred more resistance to deltamethrin than Na_V_R1, evidenced by the* Kd*T_50_ of the homozygote R2R2 (97.8 min), higher than the homozygote R1R1 (67.4 min), corroborating the hypothesis that the 1016 Ile^*kdr*^ mutation synergises with 1534 Cys^*kdr*^, providing higher levels of resistance.

The heterozygote R1R2 was intermediate (79.0 min), suggesting a synergistic effect of these two alleles. The heterozygotes with the wild-type Na_V_S or the “duplicated” Na_V_D alleles were all knocked down before 60 minutes, however with higher* Kd*T_50_ than the homozygotes SS and DD, characterizing an incomplete recessive inherence of the* kdr* alleles.

The I1011M mutation is frequent in* Ae. aegypti* Brazilian natural populations resistant to pyrethroids, especially on those where the Na_V_R2 allele is absent [30]. A selection pressure with pyrethroid in the laboratory increased the frequency of 1011M, where 100% of the insects had the mutation, but only heterozygotes were found [24] likewise in field populations [21]. This finding raised the hypothesis that I1011M mutation was part of a duplication event, which was evidenced by DNA sequencing and copy number variation qPCR assays [21]. It is of note that this Dup lineage had been originally selected from a field population and was not backcrossed with Rockefeller, differently from the process that originated R1R1 and R2R2 colonies. Although, we cannot assume the inexistence of any other resistance mechanism in the Dup lineage, its* Kd*T_50_ to deltamethrin (30 min) was only twice the Rockefeller's (14.5 min), not representing an expressive tolerance. It is possible to conclude that the 1011 Ile/Met mutation in this “heterozygous” conformation is not important for* knockdown* resistance compared to those in 1016 and 1534 Na_V_ sites. In agreement, the same aforementioned electrophysiological assays demonstrated that I1011M reduced the sensitivity of the channel to permethrin but not to deltamethrin [37].

We took advantage of a large sampling of* Ae. aegypti* in neighbourhoods from Rio de Janeiro city and surroundings [33] for exploring the frequency of the* kdr* alleles of 27 localities. The predominance of the “resistant genotypes” (R1R1, R1R2, and R2R2) ranged from 65 to 100% among the sampled localities. Additionally, the high frequency of “resistant genotypes” matches the higher levels of resistance to pyrethroids observed from South-eastern Brazilian localities [8, 30, 47]. When pooling the samples in Rio, Baixada, and Niteroi, we do not find significant difference in the genotypic frequencies between Rio and Baixada, but between Niteroi and both Rio and Baixada sites. The distinct intensity of insecticide application and an unlikely active migration of* Ae. aegypti* among these localities may reflect the observed differences in the* kdr* genetic background. A population genetic analysis based on nuclear single nucleotide polymorphisms (SNP) and microssatelites revealed low overall spatial structuring among 15 out of the same 27* Ae. aegypti* populations from Rio herein evaluated for* kdr* genotyping. The exception was the population from Paquetá island, the only which significantly differed from the other localities [33], likely due to the limited gene flow island-continent. Accordingly, samples from Paquetá presented the most divergent* kdr* frequencies and presented the highest level of the wild-type Na_V_S allele. In this island there is a preoccupation about natural and cultural heritage conservation, in a way that the employment of insecticides is supposedly better planned and controlled than in the continent. Even though still very high, the lower frequency of* kdr* alleles was therefore expected in Paquetá island. In a recent study with* Ae. aegypti* from five neighbour towns around Merida, Yucatan, and Mexico,* kdr* frequencies, in both 1016 and 1534 Na_V_ sites, significantly varied among towns. These differences were also significant in a finer scale at the block levels in two of the evaluated towns [48].

## 5. Conclusion

Several mutations in the* Na*_*V*_ gene are likely to confer pyrethroid resistance; however due to significant fitness cost they remain at very low frequencies. Other mutations conferring similar or higher levels of resistance, nevertheless with lower fitness cost, are expected to evolve and disseminate in the population, under pyrethroid selection pressure [10]. As the selection exerted by governmental campaigns and by household insecticide applications has been continuous over* Ae. aegypti*, new other mutations are likely to be emerging from the current known wild-type and* kdr* alleles and possibly conferring even higher resistance levels, as recently evidenced in the house fly* M. domestica* [49]. This highlights the importance of monitoring not only the currently known* kdr* sites by direct genotyping technics but also the strategies of whole Na_V_ gene sequencing associated with bioassays. Here we evidenced that the Na_V_R2* kdr* allele confers higher level of resistance to pyrethroid than this counterpart Na_V_R1 in* Ae. aegypti* laboratory lines with similar genetic backgrounds, also corroborating with the hypotheses of recessive inherent pattern of these* kdr* mutations. Therefore, the homozygous* kdr* genotypes, as well as the heterozygous R1R2, are likely to be the ones selected for pyrethroid resistance. Additionally, the mutation in the 1011* Na*_*V*_ site was not important for resistance in the lineage with a duplication in the* Na*_*V*_ gene, conferring a heterozygous-like aspect to this mutation. A* kdr* genotyping survey of* Ae. aegypti* from 27 distinct localities from Rio de Janeiro city and surroundings detected high frequencies of “resistant genotypes,” probably reflecting the high selection pressure exerted principally by household insecticide applications. This kind of molecular monitoring is of relevance, yet studies for unrevealing new markers related to resistance to other classes of insecticides are necessary.

## Figures and Tables

**Table 1:** List of genotypes based on SNP reactions for 1011, 1016, and 1534 Na_V_ sites of the *Aedes aegypti* laboratory lineages here evaluated.

1011	1016	1534	Genotype*∗*
Ile/Ile	Val/Val	Phe/Phe	**SS**
		Phe/Cys	**SR1**
		Cys/Cys	**R1R1**
	Val/Ile	Phe/Phe	SR3
		Phe/Cys	**SR2** + R1R3
		Cys/Cys	**R1R2**
	Ile/Ile	Phe/Phe	R3R3
		Phe/Cys	R2R3
		Cys/Cys	**R2R2**

Ile/Met	Val/Val	Phe/Phe	**DD**

*∗*The genotypes likely to occur based on the SNP genotype reactions are evidenced in bold.

**Table 2:** *Kdr* genotypic frequencies in field populations of *Aedes aegypti* from Rio de Janeiro State, considering the 1016 and 1534 Na_V_ sites.

locality	n	observed genotype frequencies						HWE test*∗*		Resistant genotypes
		SS	SR1	SR2	R1R1	R1R2	R2R2	Σ*χ*2	p^*∗*^	
Cabuçu	52	0	0,019	0,115	0,135	0,462	0,269	8,8	0,032	0,865
Cerâmica	73	0,014	0,041	0,041	0,110	0,411	0,384	0,0	1,000	0,904
Moquetá	65	0,015	0,015	0,077	0,077	0,215	0,600	31,5	0,000	0,892
Heliópolis	36	0	0,028	0,111	0,056	0,111	0,694	58,4	0,000	0,861
Jurujuba	12	0	0	0,250	0,083	0,583	0,083	8,5	0,036	0,750
Itacoatiara	24	0	0	0,167	0,208	0,292	0,333	16,3	0,001	0,833
São Francisco	16	0	0	0,250	0	0,125	0,625	3,4	0,338	0,750
Fonseca	17	0,059	0	0,294	0,118	0,118	0,412	85,5	0,000	0,647
Ponta D'Areia	14	0	0	0,286	0	0	0,714	3,4	0,064	0,714
Piratininga	37	0,081	0,054	0,108	0,270	0,189	0,297	27,8	0,000	0,757
Tubiacanga	43	0	0,070	0,047	0,186	0,163	0,535	21,3	0,000	0,884
Valqueire	31	0	0,032	0,097	0,032	0,290	0,548	10,0	0,019	0,871
Urca	20	0	0,050	0,050	0,300	0,400	0,200	1,4	0,708	0,900
Olaria	35	0	0,000	0,029	0,086	0,314	0,571	2,8	0,425	0,971
Gamboa	17	0	0	0,059	0,059	0,529	0,353	1,3	0,718	0,941
Cajú	23	0,043	0	0,043	0,087	0,087	0,739	4,4	0,224	0,913
Pavuna	34	0	0	0,118	0,118	0,265	0,500	20,7	0,000	0,882
Méier	37	0	0,054	0	0,135	0,297	0,514	6,6	0,086	0,946
Grajaú	38	0	0	0,053	0,053	0,316	0,579	4,7	0,194	0,947
Paquetá	20	0,050	0	0,350	0,250	0,150	0,200	54,6	0,000	0,600
Vaz Lobo	35	0	0,143	0,057	0,143	0,229	0,429	13,0	0,005	0,800
Jardim Guanab	32	0	0,031	0	0,094	0,375	0,500	0,8	0,841	0,969
São Cirstóvão	23	0	0	0	0,043	0,522	0,435	1,2	0,266	1,000
Humaitá	18	0	0,056	0,111	0,167	0,500	0,167	2,6	0,454	0,833
Rio Comprido	16	0	0	0	0,125	0,313	0,563	0,8	0,364	1,000
Rio das Pedras	17	0	0	0	0,118	0,529	0,353	0,2	0,618	1,000
Taquara	26	0	0	0,038	0,077	0,192	0,692	7,9	0,048	0,962

median		0	0	0,059	0,110	0,292	0,500			0,884

*∗*Hardy-Weinberg Equilibrium test.

*∗∗* Probability considering the chi-squared distribution, for one or three degrees of freedom, respectively, for three or six genotypes.

**Table 3:** Comparisons among *kdr* genotypic frequencies of *Aedes aegypti* from distinct regions of Rio de Janeiro State.

Region pair	*χ* ^2^	df	P-value
Baixada x Niterói	10.08	2	0.006
Baixada x Rio	1.79	2	0.409
Niterói x Rio	infinity	2	<0.001
Baixada x Paquetá	13.30	2	0.001
Niterói x Paquetá	2.09	2	0.351
Rio x Paquetá	infinity	2	<0.001

Baixada (Cabuçu, Cerâmica, and Moquetá ans Heliópolis), Niterói (Jurujuba, Itacoatiara, São Francisco, Fonseca, Ponta D'Areia, and Piratininga), Rio (Tubiacanga, Valqueire, Urca, Olaraia, Gamboa, Caju, Pavuna, Meier, Grajaú, Vaz Lobo, Jardim Guanabara, São Cristóvão, Humaitá, Rio das Pedras, and Taquara), and Paquetá (Paquetá island).

## Data Availability

The data used to support the findings of this study are available from the corresponding author upon request.

## Supplementary Materials

Supplementary Materials Supplementary Figure S1. Scheme of backcrosses toward selection of* Aedes aegypti kdr* lineages, highlighting the process of background homogenization between R2R2 and R1R1.* Please follow detailed explanation in the Material and Methods.* Table Spp S1. Time of knockdown profile under exposition to the pyrethroid deltamethrin (0.03%) in* Aedes aegypti* laboratory lineages with distinct genotypes for* kdr* alleles. Table Spp S2.* Kdr* allelic frequencies, considering 1016 and 1534 Na_V_ sites, in* Aedes aegypti *populations from Rio de Janeiro State.** Supp. Material S2.* Kdr *allelic frequencies in* Aedes aegypti* populations from Rio de Janeito State**. Each dot represents the frequency of the respective allele, with the confidence interval 95% amplitude indicated by the vertical bars. A = Cabuçu, B = Cerâmica, C = Moquetá, D = Heliópolis, E = Jurujuba, F = Itacoatiara, G = São Francisco, H = Fonseca, I = Ponta D'areia, J = Piratininga, K = Tubiacanha, L = Valqueire, M = Urca, N = Olaria, O = Gamboa, P = Cajú, Q = Pavuna, R = Méier, S = Grajaú, T = Paquetá, U = Vaz Lobo, V = Jardim Guanabara, W = São Cristóvão, X = Rio Comprido, Y = Humaitá, Z = Rio das Pesdras, and 2 = Taquara.
