# Supplementary material for: Levels of Resistance to Pyrethroid among Distinct kdr Alleles in Aedes aegypti Laboratory Lines and Frequency of kdr Alleles in 27 Natural Populations from Rio de Janeiro, Brazil
Source: Biomed Res Int. 2018 Jul 11;2018:2410819. doi: 10.1155/2018/2410819 (PMC6077680; doi:10.1155/2018/2410819)
Supplement: Supplementary Materials — Supplementary Figure S1. Scheme of backcrosses toward selection of Aedes aegypti kdr lineages, highlighting the process of background homogenization between R2R2 and R1R1. Please follow detailed explanation in the Material and Methods. Table Spp S1. Time of knockdown profile under exposition to the pyrethroid deltamethrin (0.03%) in Aedes aegypti laboratory lineages with distinct genotypes for kdr alleles. Table Spp S2. Kdr allelic frequencies, considering 1016 and 1534 NaV sites, in Aedes aegypti populations from Rio de Janeiro State. Supp. Material S2. Kdr allelic frequencies in Aedes aegypti populations from Rio de Janeito State. Each dot represents the frequency of the respective allele, with the confidence interval 95% amplitude indicated by the vertical bars. A = Cabuçu, B = Cerâmica, C = Moquetá, D = Heliópolis, E = Jurujuba, F = Itacoatiara, G = São Francisco, H = Fonseca, I = Ponta D'areia, J = Piratininga, K = Tubiacanha, L = Valqueire, M = Urca, N = Olaria, O = Gamboa, P = Cajú, Q = Pavuna, R = Méier, S = Grajaú, T = Paquetá, U = Vaz Lobo, V = Jardim Guanabara, W = São Cristóvão, X = Rio Comprido, Y = Humaitá, Z = Rio das Pesdras, and 2 = Taquara. [file 2410819.f1.docx]

Supplementary Figure S1. Scheme of backcrosses toward selection of *Aedes aegypti* *kdr* lineages, highlighting the process of background homogenization between R2R2 and R1R1. *Please follow detailed explanation in the Material and Methods section.*

Table Spp S1. Time of knockdown profile under exposition to the pyrethroid deltamethrin (0.03%) in *Aedes aegypti* laboratory lineages with distinct genotypes for *kdr* alleles.

| Genotype | KdT_95_ (min)* | CI95** | RR_95_^#^ |
| --- | --- | --- | --- |
| SS | 14.5 | (5.8-36.1) | 1 |
| R1R1 | 67.4 | (52.9-85.8) | 4.6 |
| R2R2 | 97.8 | (61.8-154.8) | 6.7 |
| SR1 | 34.3 | (36.4-53.9) | 2.4 |
| SR2 | 24.1 | (18.4-31.5) | 1.7 |
| R1R2 | 79.0 | (53.0-117.6) | 5.4 |
| DD | 30.0 | (24.0-42.9) | 2.1 |
| DS | 34.4 | (29.2-40.6) | 2.4 |
| DR1 | 55.1 | (24.7-122.8) | 3.8 |
| DR2 | 38.8 | (27.7-54.4) | 2.7 |

* Time required for knockdown 95% of a given lineage or population, in minutes, obtained by Probit analysis.

** 95% confidence interval

^#^ Resistance Ratio based on *Kd*T_95_, having SS as reference.

Table Spp S2. *Kdr* allelic frequencies, considering 1016 and 1534 Na_V_ sites, in *Aedes aegypti* populations from Rio de Janeiro State.

**Supp Material S2.** ***Kdr* allelic frequencies in *Aedes aegypti* populations from Rio de Janeito State**. Each dot represents the frequency of the respective allele, with the confidence interval 95% amplitude indicated by the vertical bars. A = Cabuçu, B = Cerâmica, C = Moquetá, D = Heliópolis, E = Jurujuba, F = Itacoatiara, G = São Francisco, H = Fonseca, I = Ponta D’areia, J = Piratininga, K = Tubiacanha, L = Valqueire, M = Urca, N = Olaria, O = Gamboa, P = Cajú, Q = Pavuna, R = Méier, S = Grajaú, T = Paquetá, U = Vaz Lobo, V = Jardim Guanabara, W = São Cristóvão, X = Rio Comprido, Y = Humaitá, Z = Rio das Pesdras, 2 = Taquara.

Samples were pooled into theirs respective regions, Baixada, Niterói, Rio and Paquetá. The dots represent the allelic frequency and the bars the 95%CI limits. Although Paquetá is a neighbourhood of Rio de Janeiro City, it was considered apart, since it is an island distant from the main land of both Rio and Niterói.
